# Human apurinic/apyrimidinic endonuclease (APE1) is a prognostic factor in ovarian, gastro-oesophageal and pancreatico-biliary cancers

**DOI:** 10.1038/sj.bjc.6605541

**Published:** 2010-01-19

**Authors:** A Al-Attar, L Gossage, K R Fareed, M Shehata, M Mohammed, A M Zaitoun, I Soomro, D N Lobo, R Abbotts, S Chan, S Madhusudan

**Affiliations:** 1 Department of Clinical Oncology, Nottingham University Hospitals NHS Trust, Nottingham, UK; 2 Laboratory of Molecular Oncology, Academic Unit of Oncology, School of Molecular Medical Sciences, Faculty of Medicine and Health Sciences, University of Nottingham, Nottingham, UK; 3 Department of Pathology, Nottingham University Hospitals NHS Trust, Nottingham, UK; 4 Division of Gastrointestinal Surgery, Nottingham Digestive Diseases Centre, NIHR Biomedical Research Unit Nottingham University Hospitals NHS Trust, Queen's Medical Centre, Nottingham, UK

**Keywords:** ovarian cancer, pancreatic adenocarcinoma, gastro-oesophageal cancer, immunohistochemistry, APE1

## Abstract

**Background:**

Altered DNA repair may be associated with aggressive tumour biology and impact upon response to chemotherapy and radiotherapy. We investigated whether expression of human AP endonuclease (APE1), a key multifunctional protein involved in DNA BER, would impact on clinicopathological outcomes in ovarian, gastro-oesophageal, and pancreatico-biliary cancer.

**Methods:**

Formalin-fixed human ovarian, gastro-oesophageal, and pancreatico-biliary cancers were constructed into TMAs. Expression of APE1 was analysed by IHC and correlated to clinicopathological variables.

**Results:**

In ovarian cancer, nuclear APE1 expression was seen in 71.9% (97 out of 135) of tumours and correlated with tumour type (*P*=0.006), optimal debulking (*P*=0.009), and overall survival (*P*=0.05). In gastro-oesophageal cancers previously exposed to neoadjuvant chemotherapy, 34.8% (16 out of 46) of tumours were positive in the nucleus and this correlated with shorter overall survival (*P*=0.005), whereas cytoplasmic localisation correlated with tumour dedifferentiation (*P*=0.034). In pancreatico-biliary cancer, nuclear staining was seen in 44% (32 out of 72) of tumours. Absence of cytoplasmic staining was associated with perineural invasion (*P*=0.007), vascular invasion (*P*=0.05), and poorly differentiated tumours (*P*=0.068). A trend was noticed with advanced stage (*P*=0.077).

**Conclusions:**

Positive clinicopathological correlations of APE1 expression suggest that APE1 is a potential drug target in ovarian, gastro-oesophageal, and pancreatico-biliary cancers.

DNA base excision repair (BER) is involved in the repair of bases that have been damaged by alkylation, oxidation, or ring-saturation in addition to processing deaminated bases and DNA single-strand breaks. Although there is more than one sub-pathway of BER, in most cases excision of a damaged base by a DNA glycosylase enzyme leads to the formation of a potentially cytotoxic apurinic/apyrimidinic (AP) site intermediate (Hickson *et al*, 2000). This is a target for an AP endonuclease (APE1), which cleaves the phosphodiester backbone on the 5′ side of the AP site via a hydrolytic mechanism. The major AP endonuclease in human cells, APE1 (also called previously HAP1, Ref-1, and APEX1), accounts for over 95% of the total AP endonuclease activity in most cultured human cell lines (Demple *et al*, 1991; Robson and Hickson, 1991). In addition to its DNA-repair activity, APE1 also regulates redox function (Robson and Hickson, 1991) and transcription (Bhakat *et al*, 2003; Fuchs *et al*, 2003).

Using either antisense oligonucleotides or siRNA approaches, several groups have reported that depletion of intracellular APE1 sensitises mammalian cells to a variety of DNA-damaging agents (Chen and Olkowski, 1994; Ono *et al*, 1994; Walker *et al*, 1994; Robertson *et al*, 1997; Evans *et al*, 2000; Silber *et al*, 2002). In pancreatic cancer cell lines for example, downregulation of APE1 potentiated the cytotoxicity of gemcitabine (Lau *et al*, 2004). APE1 downregulation has also been shown to block ovarian cancer cell growth (Fishel *et al*, 2008). In melanoma cell lines, APE1 downregulation led to increased apoptosis, whereas APE1 overexpression conferred protection from cisplatin- or H_2_O_2_-induced apoptosis. (Yang *et al*, 2005).

We have undertaken a drug discovery programme to isolate small-molecule inhibitors of APE1 (Madhusudan *et al*, 2005). In the previously published study, we provided the first preclinical evidence that APE1 inhibition by a small-molecule inhibitor potentiated the cytotoxicity of a panel of base-targeting agents thought to be repaired through BER (Madhusudan *et al*, 2005). To identify drug-like leads, we recently adopted an industry-standard, high-throughput, virtual screening strategy using first-generation inhibitors as templates and screened a chemical library of 2.6 million compounds. Several novel inhibitors of APE1 were identified with the ability to potentiate the cytoxicity of alkylating agents (unpublished data).

In the current study, we provide evidence that APE1 is a prognostic marker in ovarian, gastric, and pancreatico-biliary cancer. We demonstrate positive clinicopathological correlations of APE1 nuclear expression in ovarian, gastric, and pancreatico-biliary cancers, suggesting that APE1 is also a potential drug target in these tumours.

## Materials and methods

### Study design and setting

Expression of APE1 in ovarian cancer was investigated using a tissue microarray (TMA) of 157 ovarian cancer cases. Tissue was obtained from patients with primary ovarian cancer treated at Nottingham University Hospitals (NUH) between 2000 and 2007. Survival was calculated from the operation date until 1 April 2009 when any remaining survivors were censored. During the study period, patients were treated with either single agent carboplatin (65 patients (41.4%)) or platinum-based combination chemotherapy (89 patients (56.7%): 79 out of 89 patients received carboplatin and paclitaxel; 4 out of 89 received cyclophosphamide/doxorubicin/*cis*-platinum (CAP); 3 out of 89 were treated in the ICON-5 trial using paclitaxel, carboplatin plus gemcitabine, or topotecan (Bookman *et al*, 2009); and 3 out of 89 were treated according to the SCOTROC trial protocol by carboplatin, docetaxel±topotecan; Kaye *et al*, 2009). Platinum resistance was defined as patients who had progression during first-line platinum chemotherapy or relapse within 6 months after treatment.

Expression of APE1 in gastro-oesophageal cancers was investigated using two TMA sets. The first set consisted of 142 gastric/gastro-oesophageal cancer cases not exposed to neoadjuvant chemotherapy. With recent incorporation of neoadjuvant chemotherapy as a standard treatment option for operable gastro-oesophageal tumours (Cunningham *et al*, 2006), we also established a second TMA of 103 gastric/gastro-oesophageal cancer cases exposed to preoperative platinum-based chemotherapy. Tissue was obtained from patients treated at Nottingham University Hospitals (NUH) between 2001 and 2008. Survival was calculated from the date of diagnosis until 13 January 2009 when any remaining survivors were censored. During the study period, patients in the neoadjuvant arm with adenocarcinomas were treated with either neoadjuvant ECF (epirubicin (50 mg m^−2^), cisplatin (60 mg m^−2^), and continuous infusional 5-FU (200 mg m^−2^ per day)) or ECX (epirubicin (50 mg m^−2^), cisplatin (60 mg m^−2^), and capecetabine (625 mg m^−2^ p.o. b.d continuously)) chemotherapy up to three cycles prior to surgery. Patients with squamous cell carcinoma were treated with CF (cisplatin (80 mg m^−2^) and infusional 5-FU (1000 mg m^−2^ daily for 4 days); Allum *et al*, 2009) chemotherapy up to two cycles prior to surgery.

Expression of APE1 in pancreatico-biliary cancer was investigated using a TMA of 120 cases. All the participants were patients who underwent pancreatic resection (for benign or malignant pancreatic disease) between June 2001 and June 2006 at NUH Queen's Medical Centre Campus. Cores from 72 malignant tumours were selected for analysis within this study, of which 34 had pancreatic adenocarcinoma (PAC), 20 had ampullary adenocarcinoma (AAC), and 18 had cholangiocarcinoma. Only 15 patients had received adjuvant 5-FU/folinic acid chemotherapy in this cohort.

The conduct of this study was approved by the Ethics Committee of Nottingham University Hospitals.

### Construction of TMA

Tissue microarrays were constructed as described previously (Kononen *et al*, 1998). In short, area-specialised histopathologists identified and marked formalin-fixed, paraffin-embedded tissue blocks containing tumour tissue on haematoxylin- and eosin-stained slides. The marked areas in these donor paraffin blocks were used to construct the TMA. Triplicate tissue cores with a diameter of 0.6 mm were taken from the marked areas and arrayed into a recipient paraffin block using a tissue puncher/arrayer (Beecher Instruments, Silver Spring, MD, USA) as previously described (Kononen *et al*, 1998). Five-micrometre sections of the tissue array block were cut and placed on Fisherbrand Colorfrost/Plus microscope slides (Fisher Scientific, Pittsburgh, PA, USA) for immunohistochemical staining.

### Immunohistochemistry

A standard streptavidin–biotin complex method was used. Negative controls were obtained by omitting the primary antibody in each case. The tissue slides were deparaffinised with xylene and then rehydrated through five decreasing concentrations of alcohol (100, 90, 70, 50, and 30%) for 2 min each. Endogenous peroxidise activity was blocked by incubation in a 1% hydrogen peroxide/methanol buffer. Antigen retrieval was performed by microwave treatment of the slides in sodium citrate buffer (pH 6.0) for 10 min. The slides were rinsed in phosphate buffer solution (PBS) and incubated with blocking serum diluted in PBS to block non-specific staining. The slides were incubated for 1 h with the primary rabbit polyclonal anti-APE-1 antibody clone NB100-101 (Novus Biologicals Inc., Littleton, CO, USA) at a dilution of 1 : 500. After washing with PBS, sections were incubated with the secondary antibody (Vector Laboratories, Burlingame, CA, USA) for 30 min followed by the avidin–biotin complex for a further 30 min. 3–3′-Diaminobenzidine tetrahydochloride was used as chromogen. All sections were counterstained with Gill's haematoxylin.

### Evaluation of immune staining

The tumour cores were evaluated by specialist pathologists and oncologists blinded to the clinicopathological characteristics of patients. Whole-field inspection of the core was included in the assessment and intensities of staining were grouped as follows: 0=no staining; 1=weak staining; 2=moderate staining; and 3=strong staining. Cytoplasmic and nuclear staining were assessed separately for each core. Only stained malignant cells (epithelial cancer cells in ovarian cancer, ductal and acinar exocrine epithelial cells in pancreatico-billiary cancer) were included in the evaluation of staining. Stained stromal cells, inflammatory cells, and endocrine cells within islets of Langerhans were disregarded. Nuclear and cytoplasmic score were assigned separately for each core, and the mean of three cores was calculated for each sample. The results shown represent the mean score from the three cores available for each assessable patient sample. Not all cores within the TMA were suitable for immunohistochemistry (IHC) analyses due to small technical problems such that some cores were missing or lacked tumours.

### Statistical analysis

Statistical analysis of data was performed using SPSS version 15.0 for Windows (SPSS Inc., Chicago, IL, USA). Univariate analysis of associations was determined using the Pearson *χ*^2^-test. Survival rates were calculated from the time of resection until the end of the follow-up period and Kaplan–Meier curves were plotted. The statistical significance of differences between survival rates was determined using the log-rank test. Survival was censored if the patient was still alive or died of other causes. *P*-values <0.05 were identified as statistically significant. The threshold for significance was adjusted to <0.01 for subset analyses.

## Results

### Patient demographics

#### Ovarian cancer

This cohort comprised female patients with a median age of 61 years (range 33–87, mean 60 years) (Table 1a). Out of the 157 patients included in the study, 54 (34%) were dead before the end of the follow-up period. Histologically, most patients were found to have a serous cystadenocarcinoma (56%), followed by endometrioid (21%), clear-cell carcinoma (13%), mucinous cystadenocarcinoma (8%), or other types (2%). Tumours displayed poor histological differentiation in 73% of cases. The majority of tumours were classified as FIGO stage III (45%).

#### Gastro-oesophageal cancer

There were two groups of patients: those who received at least one cycle of neoadjuvant chemotherapy (neoadjuvant group) and those who underwent primary surgery (primary group) (Table 1b). There were 103 patients in the neoadjuvant group, with a median age of 63 years and 81% were male. T3 tumours were the majority, constituting 62% of cases. A total of 142 cases were in the primary group, with a median age of 74 years: 74% were male, 53% had T3 tumours.

#### Pancreatico-biliary cancer

Of the 72 patients with pancreatico-bilairy tumours, 44 patients were male and 28 were female (Table 1c). Thirty-four had PAC, 20 had AAC, and 18 had cholangiocarcinoma. The median follow-up time was 20.4 months; 35 patients (38%) died of their cancer at the time of the observation period, while only 12 patients (13%) were still alive at 5 years following the operation. The median survival was 37.8 months.

### APE1 expression and clinicopathological correlations

#### Ovarian cancer

**Figure 1:**
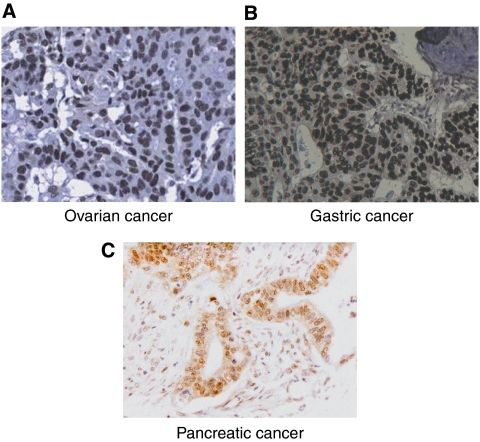
Microphotographs of ovarian cancer (**A**), gastric cancer (**B**), and PAC (**C**) showing positive nuclear APE1 expression (magnification × 200).

**Figure 2:**
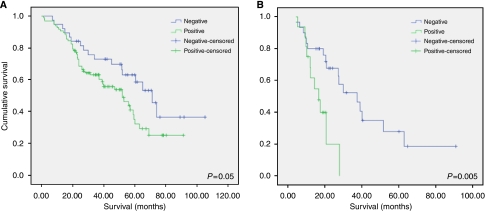
Kaplan–Meier curves showing overall survival in ovarian (**A**) and gastric (**B**) cancers according to the expression of APE1. In both cases, APE1 was associated with worse survival (log-rank test *P*-value=0.05 and 0.005 for ovarian and gastric cancers, respectively).

Apyrimidinic endonuclease-1 expression was noted both in the cytoplasmic and nuclear compartments in ovarian cancer; however, nuclear staining was more prevalent (Table 2). Of the 135 evaluable cores, 97 had nuclear expression (71.9%), but only 40 had cytoplasmic expression (29.6%). It was seen more often localised to the nucleus only (45.9%) (Figure 1A) as compared with 3.7% showing exclusively cytoplasmic staining. There was a significant difference in the level of APE1 expression among the different histological subtypes of ovarian cancer, with serous and mucinous tumours displaying nuclear APE1 more frequently than endometrioid and clear-cell carcinomas (*P*=0.006) (Table 3). Nuclear localisation of APE1 correlated with a lower likelihood of achieving optimal debulking after initial surgery (*P*=0.009), suggesting a more aggressive phenotype. This was also reflected on survival; APE1-positive cases had worse overall survival (median survival time=52 months) as compared with 71 months for APE1-negative patients. This difference was statistically significant as shown in the Kaplan–Meier graph and log-rank test (*P*=0.05) (Figure 2A). No significant correlations were seen between APE1 and FIGO stage or with histological grade.

Platinum resistance (defined as patients who had progression during first-line platinum chemotherapy or relapse within 6 months after treatment) was more frequently seen in APE1-positive cases (35.8%) as compared with APE1-negative patients (21.1%); however, this did not reach statistical significance (*P*=0.09).

#### Gastro-oesophageal cancer

In the primary-group TMAs, 41 out of 93 tumours that were stained for APE1 showed specific nuclear staining (44.1%) and 48 out of 93 (51.6%) were positive in the cytoplasm compartment as well (Table 2). In the neoadjuvant-group TMAs, 16 out of 46 tumours that were stained for APE1 showed specific nuclear staining (34.8%) (Figure 1B) and 32 out of 46 (69.6%) were positive in the cytoplasm. In the neoadjuvant group, nuclear APE1 correlated with worse overall survival in patients receiving platinum-based neoadjuvant chemotherapy (*P*=0.005) (Figure 2B). Cytoplasmic APE1 correlated with tumour differentiation (*P*=0.034). No significant correlations were seen in the primary surgery group.

#### Pancreatico-biliary cancer

The number of cancer tissue cores suitable for immunohistochemical analyses was 72, which comprised PAC (*n*=34) and AAC (*n*=20), and cholangiocarcinoma (*n*=18) (Table 2). Overall, cytoplasmic expression of APE1 was never seen in the absence of nuclear expression in either tumour or normal tissues. Nuclear staining only was seen in 44% of all periampullary tumours (32 out of 72) (Figure 1C), 35% (12 out of 34) of PACs, 60% (12 out of 20) of AACs, and 44% (8 out of 18) of cholangiocarcinomas. Absence of cytoplasmic staining was associated with vascular invasion (*P*=0.05), perineural invasion (*P*=0.007), and a trend towards advanced stage (*P*=0.077) and poor differentiation (grade-3 poorly differentiated adenocarcinoma) (*P*=0.068).

## Discussion

Overall prognosis of advanced melanoma, ovarian, gastro-oesophageal, and pancreatico-biliary cancer remains poor. New treatment strategies are urgently required to address this largely unmet need. Cytotoxic agents (such as alkylating agents and platinum compounds) and ionising radiation used in the treatment of these tumours induce DNA damage (such as base damage and others) in cells. Cancer cells proficient in DNA repair are able to repair such DNA damage and continue to survive. This is a significant cause for therapeutic resistance and impacts negatively on patient outcomes. Pharmacological inhibition of DNA repair is likely to enhance the cytotoxicity in cancer cells and improve tumour response in patients. APE1 is a key multifunctional protein essential for DNA BER, in addition to redox regulation of transcription factors and transcriptional regulation. It is widely accepted that nuclear expression of APE1 is involved in DNA repair, whereas cytoplasmic expression may be involved in redox/transcriptional regulation.

In the current study we have demonstrated the potential APE1 expression has as a negative prognostic factor in cancer patients. Nuclear expression of APE1 was frequently seen in ovarian, gastro-oesophageal, and pancreatico-biliary cancers. In ovarian cancer, nuclear APE1 expression was seen commonly in serous and mucinous tumours (*P*=0.006). In addition, sub-optimal debulking and residual tumour was seen more frequently in tumours with nuclear expression of APE1, implying aggressive tumour biology (*P*=0.009). In fact, this was reflected in poor overall survival in patients (*P*=0.05). Interestingly, we also found evidence that APE1 expression may predict platinum resistance in patients, although this did not reach statistical significance (*P*=0.09). The negative prognostic impact was also seen in gastro-oesophageal cancer where patients who received neoadjuvant platinum-based chemotherapy and whose tumours showed APE1 nuclear expression did significantly worse as compared with non-expressors (*P*=0.005). Our study is consistent with recent preclinical observations using melanoma cell lines where APE1 overexpression conferred protection from cisplatin (Yang *et al*, 2005). Moreover, in lung cancer, blocking APE1 function potentiated cisplatin cytotoxicity (Wang *et al*, 2009). These studies imply that APE1 is involved in the repair of damage induced by platinating agents, and current evidence suggests that the protective effect may be contributed by the DNA repair as well as the redox-regulatory activity of APE1. The negative prognostic significance of APE1 has also been recently demonstrated in osteosarcoma (Wang *et al*, 2004) and head and neck cancer (Koukourakis *et al*, 2001).

Apyrimidinic endonuclease-1 exhibits complex and heterogeneous staining patterns. Although the biological relevance of the compartmentalisation of APE1 is not understood, its complexity suggests that APE1 localisation in cells is tightly regulated. In addition, as the nuclear expression of APE1 is involved in DNA repair and the cytoplasmic expression may be involved in redox/transcriptional regulation, it is speculated that the compartmentalistion may reflect distinct functions of APE1 in cells. The altered subcellular localisation of APE1 in pancreatico-biliary cancers observed in our study is distinct to observations in other tumours. Nuclear staining only was seen in 44% of all periampullary tumours. Combined nuclear and cytoplasmic expression was seen in 25% of tumours. Cytoplasmic expression of APE1 was never seen in the absence of nuclear expression. Absence of cytoplasmic expression correlated to adverse prognostic features. This is in contrast to tumours such as breast cancer. In normal breast tissue, APE1 localisation is predominantly nuclear, whereas in breast carcinomas, nuclear, cytoplasmic, and nuclear/cytoplasmic stainings were observed (Kakolyris *et al*, 1998; Puglisi *et al*, 2002). Furthermore, while nuclear localisation was associated with good prognostic features (being related to better differentiation, low angiogenesis, and negative lymph node status), cytoplasmic and combined nuclear/cytoplasmic localisation were associated with poor prognostic factors, such as angiogenesis together with node and p53 positivity (Kakolyris *et al*, 1998; Puglisi *et al*, 2002). A dysregulation in nuclear *vs* cytoplasmic ratio towards increased cytoplasmic staining was also observed in thyroid carcinomas (Tell *et al*, 2000) and prostate cancer (Kelley *et al*, 2001).

In conclusion, we have shown prognostic implications of APE1 in ovarian, gastro-oesophageal, and pancreatico-biliary cancer. Moreover our study suggests that APE1 is a potential drug target in these tumours. The recent evidence of BER modulation using PARP inhibitors that have shown promise in recent clinical trials in ovarian and breast cancer (Lord and Ashworth, 2008), implies that APE1 inhibitors may have similar clinical application in patients.

## Figures and Tables

**Table 1a:** Patient demographics and pathological features for Ovarian cancer

**Characteristics**	**Frequencies**	**Percentages (%)**
*Pathology*		
Serous cystadenocarcinoma	88	56
Endometrioid	33	21
Clear cell carcinoma	21	13
Mucinous cystadenocarcinoma	12	8
Other	3	2
		
*Grade*		
1	20	13
2	23	14
3	114	73
		
*Residual tumour*		
None	88	56
Present	69	44
		
*Stage*		
IC	44	28
II	21	13
III	71	45
IV	21	14
		
*Chemotherapy*		
Carboplatin monotherapy	65	41
Carboplatin combination therapy	89	57
No chemotherapy	3	2
		
*Platinum sensitivity*		
Sensitive	104	66
Resistant	50	32
Unknown	3	2
		
*Relapse status*		
Relapsed	75	48
Relapse-free	75	48
Unknown	7	4
		
*Survival status*		
Living	103	66
Dead	54	34

**Table 1b:** Patient demographics and pathological features for Gastro-oesophageal cancer

**Characteristics**	**Primary surgery arm (*n*)**	**Percentage (%)**	**Neoadjuvant arm (*n*)**	**Percentage (%)**
Patients (*n*)	142		103	
Median age	74		63	
				
*Sex*				
Male	105	73.9%	83	81%
Female	37	26%	20	19%
				
*T stage*				
T1	14	9.8%	4	3.8%
T2	48	33.8%	24	23.6%
T3	75	52.8	64	62%
T4	5	3.5%	9	8.6%
TX			2	2%
				
*N stage*				
N0	33	23.2%	29	28%
⩾N1	109	76.8%	74	72%
				
*M stage*				
M0	140	98.5%	103	100%
M1	2	1.4%	—	
				
*Vascular invasion*				
No	44	40%	57	55%
Yes	98	60%	46	45%
				
*Perineural invasion*				
No	66	46%	15	15%
Yes	76	54%	88	85%
				
*Status*				
Alive	54	38%	47	46%
Dead	87	62%	56	54%

**Table 1c:** Patient demographics and pathological features for Pancreatico-biliary cancer

**Characteristics**	**No of patients**	**% of total number of recorded cases**
*Age (years)*		
<40	0	
41–60	28	38.9
>60	44	61.1
		
*Sex*		
Male	44	61.1
Female	28	38.9
		
*Grade*		
Poor	14	19.4
Well/moderate	49	68.1
Unknown	9	12.5
		
*Lymph node status*		
Not involved	17	23.6
Involved	46	63.9
Unknown	9	12.5
		
*Vascular invasion*		
Negative	33	45.8
Positive	37	51.4
Unknown	2	2.8
		
*Perineural invasion*		
Negative	32	44.4
Positive	39	54.2
Unknown	1	1.4
		
*T stage*		
0	1	1.4
1	7	9.7
2	20	27.8
3	40	55.6
4	3	4.2
Unknown	1	1.4
		
*Outcome*		
Alive	26	36.1
Died of disease	30	41.7
Died of other cause	16	22.2
		
*Site*		
Pancreatic	24	38.7
Ampullary	20	32.3
Cholangio	18	29

**Table 2:** The distribution of nuclear APE1 expression in ovarian, pancreatico biliary, and gastric cancers (primary and neoadjuvant series)

	**Ovarian**	**Pancreatic**	**Gastric (primary)**	**Gastric (neo-adjuvant)**
Positive	97 (71.9%)	32 (44.4%)	41 (44.1%)	16 (34.8)
Negative	38 (28.1%)	40 (55.6%)	52 (55.9%)	30 (65.2%)
				
Total	135 (100%)	72 (100%)	93 (100%)	46 (100%)

**Table 3:** Cross-tabulation of APE1 expression in ovarian cancer with histopathological subtypes

**APE1 nuclear ovarian cancer pathological subtypes – Cross-tabulation Pathological subtypes**						
	**Serous**	**Mucinous**	**Endometroid**	**Clear cell**	**Other**	**Total**
*APE1 nuclear*						
*Negative*						
Count	14	3	12	9	0	38
%	17.9%	30.0%	42.9%	52.9%	0%	28.1%
						
*Positive*						
Count	64	7	16	8	2	97
%	82.1%	70.0%	57.1%	47.1%	100.0%	71.9%
						
*Total*						
Count	78	10	28	17	2	135
%	100%	100%	100%	100%	100%	100%

Abbreviation: APE1= apurinic/apyrimidinic endonuclease-1.

A strong correlation was found (*P*=0.006).
